# Growing empathy through art therapy, life story, and relationships: experiential learning in adult day services

**DOI:** 10.3389/fpsyg.2024.1489344

**Published:** 2024-12-18

**Authors:** L. Blake Peeples, Benjamin C. Thompson, Jackie B. Tucker, LaDerrick Smith, Amy Brown, Keisha D. Carden, Anne Halli-Tierney, Angel C. Duncan, Neelum T. Aggarwal, Jessica Y. Allen, Rebecca S. Allen, Daniel C. Potts

**Affiliations:** ^1^Department of Psychology, The University of Alabama, Tuscaloosa, AL, United States; ^2^Department of Psychological and Brain Sciences, The University of Louisville, Louisville, KY, United States; ^3^Alabama Research Institute on Aging, The University of Alabama, Tuscaloosa, AL, United States; ^4^Cognitive Dynamics Foundation, Tuscaloosa, AL, United States; ^5^VA Maryland Health Care System, Baltimore, MA, United States; ^6^Departments of Neurological Sciences, Rush Alzheimer’s Disease Center, Rush University Medical Center, Chicago, IL, United States; ^7^Alabama Life Research Institute, The University of Alabama, Tuscaloosa, AL, United States

**Keywords:** bringing art to life, persons with dementia, empathy, attitudes, community service

## Abstract

**Introduction:**

Empathy is a fundamental element of high-quality healthcare, though it has been shown to be in decline among medical students and residents. Appeals have therefore been made for the development of evidence-based empathy-enhancing experiential learning and training models. Bringing Art to Life (BATL) is a service-learning program designed within experiential learning pedagogy for psychology and pre-healthcare students. Intergenerational relationships are fostered with people with dementia through art therapy and life story/narrative at a community-based adult day services center.

**Methods:**

In this sequential mixed methods study, quantitative data were collected via electronic surveys of students in this course compared with students in didactic introductory psychology or psychology and aging courses. Survey measures included empathy, mindfulness, positive and negative affect, future time perspective, and attitudes toward older adults and working with people with dementia. Weekly BATL student journals submitted as part of their course requirements were analyzed using qualitative content analysis.

**Results:**

Within-subjects analyses of variance revealed increases in empathy and positive affect, broadened time perspective, and improved attitudes toward older adults and people with dementia among students in the BATL course compared with other undergraduate students. Analysis of BATL student journals supported and deepened understanding of these findings, with themes including attitude change, relationship building fostering existential awareness and self-development, art fostering social interactions, and perceived program effectiveness.

**Discussion:**

The findings suggest that BATL strongly supports increased empathetic attitudes and decreased stigma of aging in psychology and pre-healthcare students. Intergenerational expressive arts-based programs like BATL should be implemented in healthcare education to enhance empathy and improve attitudes toward aging and dementia care.

## Introduction

**Figure 1:**
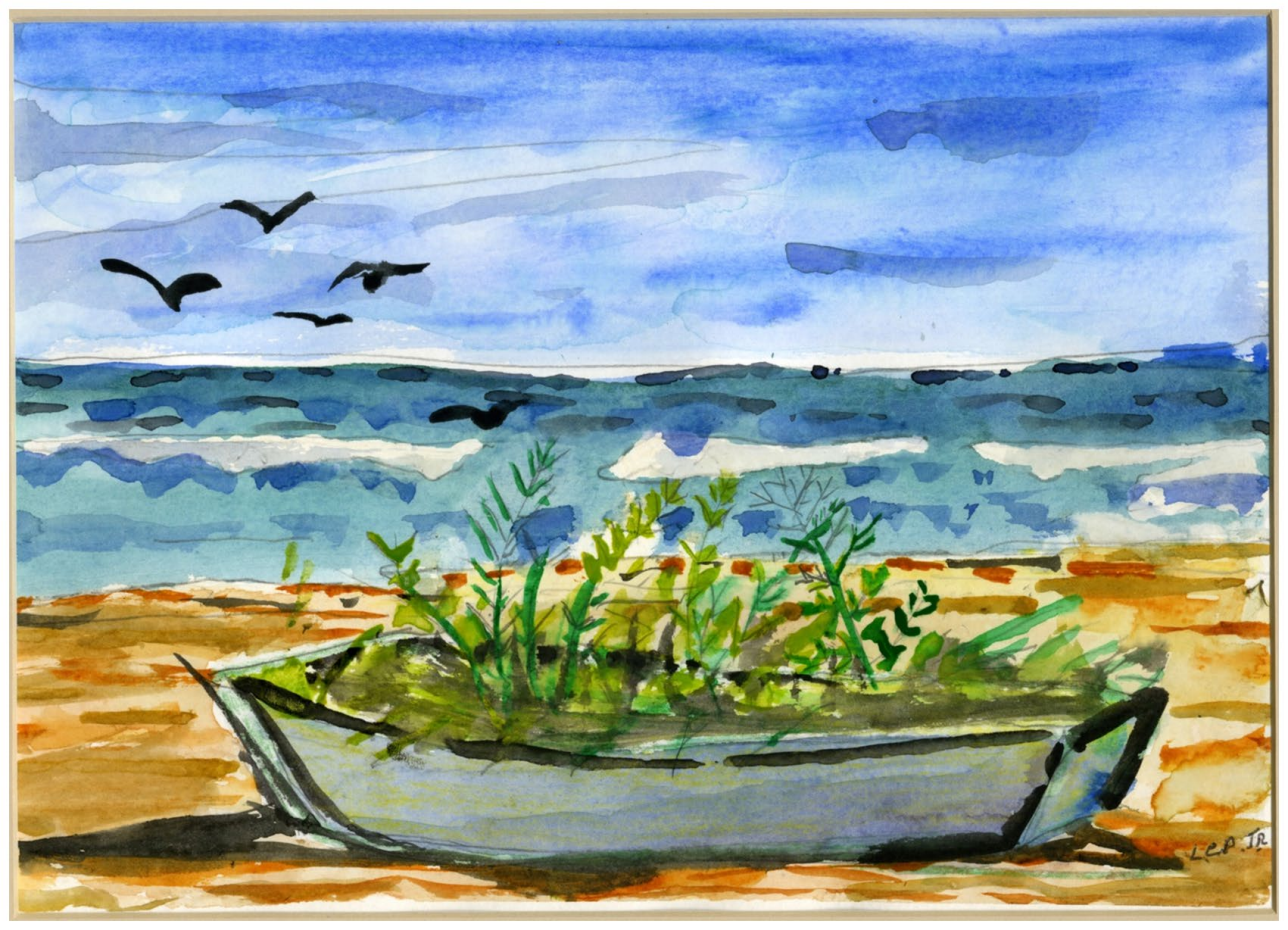
Artwork created by Lester E. Potts, Jr., a person living with dementia who was a client at Caring Days Adult Day Services Center in Tuscaloosa, Alabama.

**Figure 2:**
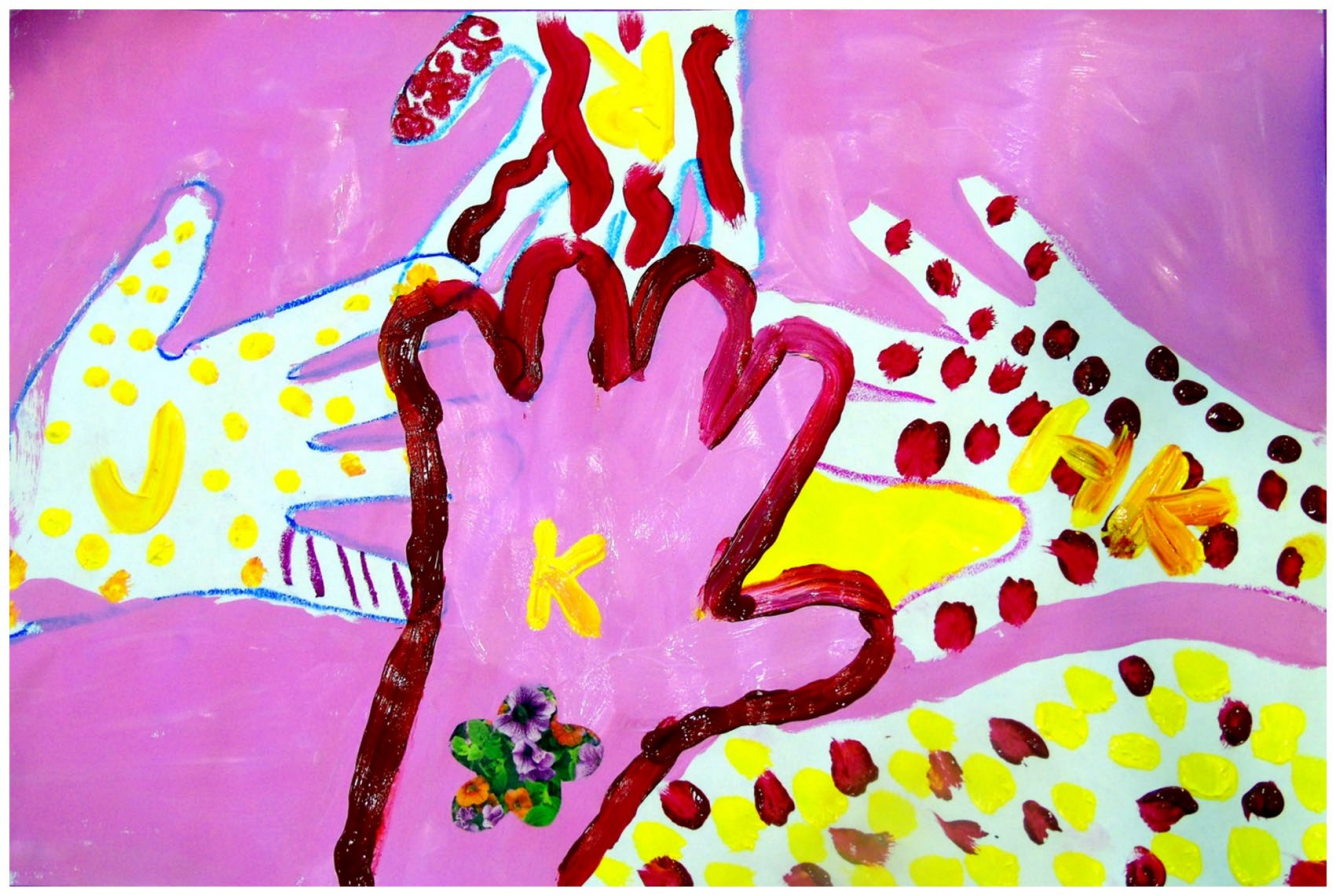
Collaborative artwork created by Katie W., a person living with dementia who was a client at Caring Days Adult Day Services Center in Tuscaloosa, Alabama, and her BATL student partners.

*Bringing Art to Life* (BATL) is a service-learning program based in experiential learning pedagogy (Leary and Sherlock, 2020) developed by the Cognitive Dynamics Foundation in memory of Lester E. Potts, Jr., an artist who had Alzheimer’s disease (Figure 1). It is offered as a junior seminar psychology writing course at [blinded for review]. Its primary purpose is to honor and validate people with dementia and other cognitive disorders through art therapy, other expressive arts, and life story/narrative. Additional goals include facilitating the development of intergenerational, multicultural relationships and growing empathy, compassion, knowledge, and self-awareness in students via experiential educational paradigms. Stated goals of the course are to lessen stigma, provide respite for care partners, and lay a foundation for ongoing engagement and enrichment of students, people with dementia, and their care partners in the broader community (Potts, 2022). Case studies and several trials suggest that art therapy demonstrated significant gains among people with dementia including improved quality of life, engaged attention, experienced pleasure, and improved neuropsychiatric symptoms, social behavior, and self-esteem (Chancellor et al., 2014; Emblad and Mukaetova-Ladinska, 2021; Lam et al., 2020). These expressive arts interventions also reduce stigma (Bienvenu and Hanna, 2017) (Figure 2).

### Anti-stigma

Stigma is referred to as negative attitudes and biases held about persons that could be result of some diagnosis (Dinos et al., 2004). A lack of knowledge regarding dementia can cause an unwarranted stigma toward persons living with the disease, illustrated by a theory known as stereotype embodiment (Levy, 2009). The theory suggests stereotypes are manifested when their understanding is driven from external sources which create self-definitions that impact functioning and health. Stigma-driven thoughts held by individuals can often lead to negative response, prejudice, and/or avoidance from a population (Corrigan and Watson, 2002). Therefore, it is important for emerging adults to gain the necessary knowledge of dementia to reduce the stigma surrounding it.

Emerging adults may particularly benefit from experiences that aim to reduce stigma for people with dementia. Prior research suggests that education, awareness, and experience with people with dementia may increase positive outcomes. For example, previous research has shown that emerging adults showed statistically significant levels of dementia stigma, whereas professional care-workers showed more dementia-positive attitudes (Kane et al., 2018).

In a 2004 anti-stigma study regarding people with dementia, only 4% of lay persons believed that people with dementia were at fault for their behaviors. Rather, the participants were more inclined to help people with dementia and see them as individuals in need of guidance (Werner and Davidson, 2004). To further add to this, a 2018 study used an intergenerational choir participation intervention with college students, persons with Alzheimer’s disease, and their relatives. The study resulted in reduced social isolation of persons with Alzheimer’s and their families and stigma among college students (Harris and Caporella, 2018).

Notably, seeing people with dementia as people who need constant care can lead to an attitude that people with dementia are helpless, also known as infantilization (Jongsma and Schweda, 2018). People do not want to be infantilized or seen as helpless, so they will often disregard the signs of early-stage dementia to ignore the pervasive cultural trope (Jongsma and Schweda, 2018). Evidence-based, experiential learning programs can address these nuances through targeted education and person-to-person interactions. Research has shown that in addition to education, exposure to and experience with people with dementia matter. For example, stigma was lower in persons who had experience with people with dementia, who were younger and more educated about dementia, and who thought treatment was available for dementia (Cheng et al., 2011). Thus, there is a need for public health interventions to alleviate dementia stigma. BATL is one such paradigm, working to decrease stigma and fear while highlighting the personhood of people with dementia by experientially increasing empathy.

### Empathy

Empathy is defined as “understanding a person from his or her frame of reference rather than one’s own, or vicariously experiencing that person’s feelings, perceptions, and thoughts” (*APA Dictionary of Psychology*, American Psychological Association, 2018). There is a robust literature available regarding personhood and empathetic approaches in dementia care. Firstly, Thomas Kitwood shifted the perspective from a biomedical view to one focused on the lived experience of people with dementia, with techniques such as dementia care mapping (Kitwood, 1997). Additionally, he and Kathleen Bredin focused on a person-centered approach emphasizing life quality and dignity rather than deficit and loss (Kitwood and Bredin, 1992). Kitwood’s book, *Dementia Reconsidered* (Kitwood, 1997), and his work with Bredin are foundational components for the field still used in clinical training for dementia care (Mitchell and Agnelli, 2015).

The personhood perspective differs greatly from the perspective of dementia as a “social death” (Sweeting and Gilhooly, 2008), a concept that is rooted in deficit-based, biomedical mechanisms often attributed to earlier works like those of Mace and Rabins (1981). For example, studies on dementia care models (e.g., Kitwood, 1997) highlight the need for sustained empathy and ongoing education in dementia care. Kitwood’s work demonstrates that dementia care should prioritize understanding and affirming the person’s identity and unique preferences. Sustained empathy and continued education provide the needed foundational skills to adapt to changing needs and maintain a respectful, individualized approach, thereby reducing “social death” risks for people with dementia. Research related to resisting societal disenfranchisement is one example of how researchers have challenged the narrative of “Alzheimer’s patients” to be more identity-centered (Beard and Fox, 2008).

*Bringing Art to Life* aims to expand on the work of colleagues, such as Beard and Fox, through its building of identity-centered intergenerational relationships between emerging adult students and people with dementia. Research suggests that increased training and understanding of emotive empathy in caregivers improves the level of care provided to older adults living with neurological diseases through improved emotional intelligence (Maximiano-Barreto et al., 2022). The lack of empathy training correlates with a lack of proper care for people with dementia, as these individuals are not commonly included in creating their own care plan, and most choices are made by a family member or other primary caregiver(s) (Wackerbarth, 1999). Although it is important for these family members and caregivers to provide guidance, previous research shows that people with dementia want to be part of their medical decisions, and physicians found their participation beneficial to providing people with dementia with quality care (Iezzoni et al., 2021). Institutionalized people with dementia (those residing in nursing homes, specialty care assisted living facilities, and other outlets of dementia care services) acknowledged their preference for individualized and person-centered care, which emphasizes the need for close bonds grounded on empathy (Wehrmann et al., 2021). Poor understanding of individualized quality care for people with dementia can lead to caregiver burnout (De Alves et al., 2019) and reduced empathy. In contrast, BATL creates an environment in which the focus on personhood and experiential acquisition of knowledge provides opportunities to gain empathy for people with dementia.

### Didactic and experiential components of BATL and course assignments

Few educational opportunities exist for emerging, college-aged adults (about age 18–29) to understand the lived experience of people with dementia (Peisachovich et al., 2023; Arnett, 2007; Reifman et al., 2007), and this lack of knowledge and awareness may contribute to lost opportunities for compassionate care of, and interactions with, such persons. Currently, the BATL program is being implemented across multiple sites with somewhat varying target participants, both in terms of learners and points of service delivery for people with dementia. In this manuscript, we describe an evaluation of the seminal BATL program involving college students and people with dementia in adult day service programs. Students undergo several weeks of education and training during a 16-week semester while simultaneously being paired with people with dementia in the treatment setting.

Students assist in group art therapy sessions once a week under the direction of an art therapist, and interact with their peers, people with dementia, and the art therapist about both the art that is produced and the creative process itself. Art therapy directives (activities) planned by the art therapist focus on several of life’s most cherished aspects, such as family, food and childhood. Examples of these directives include “Your Grandmother’s Breakfast” (prompt: “Think of a typical breakfast that you would have enjoyed with your grandmother and create art to represent that breakfast.”), “Hands Directive” (prompt: “Trace your hands in a favorite color and create an artistic representation of something unique about yourself.”), or the “Shaving Cream Art Directive” (participants and students spread a layer of shaving cream into an aluminum pan, add droplets of food coloring, swirl with a Q tip, blot with watercolor paper, then rake off the shaving cream with a straight edge to reveal marbled artistic designs). The art over which BATL students and people with dementia interact is thus produced.

Students are trained in effective communication methods for interacting with people with dementia. Mindfulness training is provided primarily as a means of enhancing the relational impact of the art therapy sessions through better listening, enhanced capacity for compassion and empathy, increased self-awareness, and as a check against prejudgments and emotional reactivity. It also may provide students with a new lens to engage discomfort, a potentially beneficial skill supporting wellness later in their lives and careers. Several student creative writing assignments and weekly journal entries about the art therapy experience and interactions with people with dementia across the semester require students to reflect experientially on course themes.

Didactic course content includes comprehensive lectures from neurologists about the neuroscience of memory, Alzheimer’s disease, and other dementias, including topics such as epidemiology, pathophysiology, common clinical manifestations and their pathophysiological correlations, diagnosis and imaging, methods of cognitive assessment, prevention, treatment, and updates on research related to racial/ethnic, health disparities and inequity in dementia care. Lectures on the history, theory, and practice of art therapy and other expressive arts therapies and a discussion of art therapy directives are offered as well as lectures on caregiving, aging, and mindfulness. Additionally, students participate in simulated, first-person, embodied dementia experiences via the Virtual Dementia Tour from Second Wind Dreams and virtual-reality training modules from Embodied Labs. Prior research has demonstrated effectiveness of the Virtual Dementia Tour, indicating that students showed significant improvements in empathy and understanding of the lives of people with dementia after training (Peng et al., 2020). Similarly, virtual reality training modules from Embodied Labs have demonstrated efficacy among medical students, who reported significant increases in understanding of the needs of people with dementia, the needs of a caregiver of someone with dementia, and the impact dementia can have on the entire family (Bard et al., 2023).

When discussing components of BATL, it is integral to highlight the importance of expressive arts programming to this intergenerational program. The current project emphasizes the need for intergenerational, experiential opportunities, such as BATL, for emerging adults and pre-healthcare students through using the mechanism and depth of expressive arts therapies. The art therapy component of BATL allows for a tangible focus and scaffold on which to build a sense of relatedness between students and people with dementia that they might not otherwise recognize. Throughout the many years of this course, students first display feelings of hesitancy and nervousness about meeting people with dementia. However, as the course continues it is clear that the collaborative art activities create a common ground that takes away feelings of wariness, nurturing a greater natural connection.

There is an extensive literature on the use of expressive arts interventions for people with dementia, highlighting quality of life over medical outcomes. This literature further highlights the need for expressive arts programming in intergenerational learning settings not only for students, but also for people with dementia. A shift in focus from behavioral issue reduction to meaningful interaction is highlighted in several studies by means of arts activities, personalized music interventions and group singing, and expressive arts in general (Gerdner, 2000; Jones and Sutherland, 2005; Noice and Noice, 2006; Creech and Hallam, 2013; Creech et al., 2013). Attention to this literature can deepen the understanding and necessity for creative interventions as a means of fostering connection and life story for people with dementia.

### Need for study

The current sequential mixed methods study aims to understand the potential impact of the BATL experiential learning course on emerging adults’ empathy, mindfulness, and attitudes toward older adults, people with dementia, and interest in community service and health-related careers. Across seven semesters, college students enrolled in BATL completed surveys and were compared with students enrolled in didactic Introductory Psychology or a 300-level Psychology and Aging course. Attitudes toward older adults, individuals with dementia, community service and additional outcomes including positive and negative affect, time perspective, psychological flexibility, mindfulness, and empathy were measured via online surveys at the beginning and end of the semester. It was expected that students enrolled in the experiential BATL course would, as measured via surveys, demonstrate more positive attitudes toward older adults and people with dementia as well as improved emotional functioning across a semester in comparison with students in Intro Psychology or Psychology and Aging, as indicated by significant time by course interaction terms.

In addition to quantitative surveys, students enrolled in BATL wrote qualitative weekly journal entries as a course requirement, and these entries were coded for emergent themes. Qualitative content coding of BATL student journals are a main source of data explanation that provide an illustrative and rich narrative. Ultimately, qualitative description of student journals complements and extends the quantitative data and is the best approach for qualitative data of the present kind, where “straight descriptions” of experiences are warranted (Sandelowski, 2000). Moreover, numbers are used in descriptive qualitative research to establish the significance, document what is known, and generate meaning from the qualitative student journal data (Sandelowski, 2001).

## Methods

For quantitative comparison analyses, participants were 367 students (*mean* age = 19.36, *SD* = 1.44, Range 17–27 years; 80% women) recruited from three different psychology courses across seven semesters at one large university in the southeastern United States. Students were required to be in the Introduction to Psychology course (*N* = 242), a 300-level Psychology of Aging course (*N* = 100), or the 300-level BATL course (*N* = 24). A purposive sampling method was applied; students self-selected into each of these courses (Palinkas et al., 2015). Students enrolled in the experiential BATL course also completed weekly journal entries as a course requirement. Journal entries were collected across seven semesters between Spring 2015 and Spring 2018, guided by a topic list created by the instructor of the course, blinded for review. The journal topics revolved around the experiences students had in each previous week’s session of art therapy and interactions with the people with dementia at the adult day service center.

### Procedure

The study was ethically approved by the [blinded for review] Institutional Review Board. Students enrolled in one of the courses described were provided with an online consent form stating that participation was voluntary, and they could skip any questions or discontinue participation at any time. Additionally, BATL class members provided 10 journal entries across each semester to capture first-person descriptions of student experiences. There were no inclusion or exclusion criteria other than enrollment in one of the courses and providing survey data at the beginning and end of the semester of enrollment.

### Quantitative measures

In addition to demographics all participants completed the following measures.

*The Student Assisted Independent Living* (SAIL; Pillemer and Schultz, 2002). This scale is a 20-item attitudinal scale rated on a seven-point Likert scale ranging from “*strongly disagree*” to “*strongly agree*” measuring four dimensions of attitudes toward individuals with dementia (i.e., older adults, community service, older adults with dementia, and working with geriatric patients and individuals with dementia). Yamashita et al. (2011) reported good reliability and validity with Chronbach’s *α*’s ranging from α = 0.64 to α = 0.76.

*The Positive and Negative Affect Schedule* (PANAS; Watson et al., 1988) measures students’ experiences of positive and negative emotions in the present moment. The questionnaire has a total of 20 items, with each item rated on a five-point Likert scale ranging from “*very slightly or not at all*” to “*extremely*.” The PANAS has good internal consistency for the positive affect (PA) Cronbach’s α = 0.84 and for the negative affect (NA) Cronbach’s *α* = 0.87 scales. The two scales have good discriminate validity as they correlate −0.09.

*The Future Time Perspective Scale* (FTPS; Carstensen and Lang, 1996) measures students’ experiences of time perspective related to an individual’s motivation/approach to their own life, with a total of 10 items, each rated on a seven-point Likert scale ranging from “*very untrue*” to “*very true*.” Individuals who score below 50 are said to view time as limited, while those who score above 50 have a more open time perspective. A recent systematic review and meta-analysis reports a mean Cronbach’s α = 0.84 for this scale (Kooij et al., 2018).

*The Mindful Attention Awareness Scale* (MAAS; Brown and Ryan, 2003) measures students’ trait-like experiences of attention and awareness. The questionnaire has a total of 15 items, with each item rated on a six-point Likert scale ranging from “*almost always*” to “*almost never*.” MacKillop and Anderson (2007) reported Cronbach’s α indicated good internal reliability, α = 0.89.

*The Empathy Quotient Short Form* (EQ; Lawrence et al., 2004; Muncer and Ling, 2006) assesses three facets of empathy: cognitive empathy, emotional reactivity and social skills. The Lawrence et al. version of the scale was made with 28 items and then shortened to a 15-item version by Muncer and Ling. The scale is a self-report measure designed to capture empathy in adults, with each item rated on a four-point Likert scale ranging from “*strongly in disagreement*” to “*strongly in agreement*.” Cronbach’s α for the short form is α = 0.88 (Wakabayashi et al., 2006).

### Qualitative methods and analysis

A qualitative analysis of student journals was completed using the Framework approach, as it is a systematic approach of analyzing qualitative data which aims to uncover practice-oriented findings (Green and Thorogood, 2004). This approach utilizes five stages: (a) familiarization; (b) identifying a thematic framework; (c) indexing; (d) charting; and (e) mapping and interpretation. For the familiarization stage, researchers read all transcribed interviews thoroughly. From the initial read through, each member of the qualitative analysis team (LBP, BCT, JBT, KDC, and RSA) independently developed a framework based on all journal entries for a particular individual in each of two different semesters to develop the initial thematic framework. The primary qualitative coders (LBP, BCT, and JBT) all met and discussed the categories uncovered until a final consensus was reached to create a final framework. The indexing stage allowed the thematic framework to be applied to all data. Tables were then created for each of the identified themes, including participant quotes, to allow a visual aid to better organize the data and to recognize and establish patterns.

Rigor, trustworthiness and an awareness of reflexivity, credibility, and believability (Creswell, 2014; Russell and Gregory, 2003) were increased by directly examining reflexivity, or what the coder brings to the coding of qualitative data, through the use of investigator triangulation (Archibald, 2016). This investigator triangulation peer review helps to keep investigators’ interpretations in check and support basic awareness of potential bias while facilitating solid evidence for the interpretation of the data (Valencia, 2022; Holt and Thorpe, 2007). Discrepancies were discussed until consensus was reached, with any areas of disagreement in the primary coding team were resolved by the two senior qualitative experts (KDC, RSA).

## Results

### Participant demographics

For the quantitative, pre-post analyses, participants were recruited from only four semesters. Descriptive characteristics of the sample are presented in Table 1 and data for all surveys at the beginning and end of semesters for each class are presented in Table 2. A one-way ANOVA revealed the mean age of students varied depending on the course in which they were enrolled, *F*(2,365) = 218.78, *p* < 0.001, with Introduction to Psychology students being significantly younger than BATL students who also were slightly younger than Psychology of Aging students (see Table 1). Student self-reported gender differed across the three courses, with a higher percentage of women in the BATL course. Experience interacting with older adults with dementia also significantly differed, indicating a self-selection bias for students enrolled in Psychology of Aging and especially BATL, wherein students in these courses reported greater prior interactions with people with dementia, *Χ^2^*(2) = 20.77, *p* < 0.001. Self-reported prior volunteerism in nursing homes or in intergenerational service-learning experiences did not differ significantly.

**Table 1:** Student participant demographics.

Survey	Intro Psych (*N* = 242)	Psych and Aging (*N* = 100)	BATL (*N* = 24)
Age***	18.62 (0.84)	21.01 (1.22)	19.96 (1.08)
Sex assigned at birth (% women)*	77%	86%	92%
Experience with persons with dementia***	33%	43%	79%
Volunteer in nursing homes	39%	43%	58%
Experience with intergenerational program	12%	5%	8%

For age, means are displayed with standard errors in parentheses. For all other variables, the percentage of students in class endorsing the experience is displayed.

*p < 0.05, **p < 0.01, ***p < 0.001.

**Table 2:** Beginning and end-of-semester outcomes by course.

Survey	Intro Psych Pre (*N* = 242)	Intro Psych Post (*N* = 242)	Psych and Aging Pre (*N* = 100)	Psych and Aging Post (*N* = 100)	BATL Pre (*N* = 24)	BATL Post (*N* = 24)
Attitudes (SAIL)						
Older adults**	17.47 (1.88)	18.18 (1.99)	17.30 (1.57)	18.07 (1.39)	17.83 (1.63)	18.67 (1.71)
Persons with dementia***	12.08 (2.28)	11.52 (1.23)	12.84 (2.71)	12.54 (2.57)	14.25 (1.68)	15.55 (1.99)
Community service**	24.98 (2.74)	19.24 (1.53)	25.17 (3.08)	25.47 (2.92)	27.54 (2.48)	28.67 (2.88)
Empathy***	43.42 (4.80)	39.74 (2.83)	43.72 (4.68)	44.40 (4.55)	47.20 (4.38)	48.15 (3.72)
Positive affect***	33.36 (6.42)	24.33 (3.64)	32.25 (7.14)	33.52 (7.22)	38.45 (4.50)	39.82 (3.55)
Negative affect***	24.33 (7.22)	27.15 (3.92)	22.41 (6.21)	22.02 (6.98)	18.37 (4.85)	17.83 (5.11)
Time perspective*	54.50 (8.72)	34.33 (3.54)	55.61 (8.97)	54.34 (9.52)	58.71 (5.04)	59.71 (7.77)
Psychological flexibility	22.84 (10.17)	20.25 (6.50)	22.59 (7.71)	20.77 (8.94)	15.57 (6.31)	14.79 (7.09)
Mindfulness	3.86 (0.86)	3.67 (0.61)	3.86 (0.77)	3.88 (0.78)	4.05 (0.79)	3.99 (0.80)

Means are displayed with standard errors in parentheses. On the student assisted independent living (SAIL) scale, scores ranged from 7 to 49 for attitudes toward older adults, 8 to 56 for attitudes toward community service, and 5 to 35 for attitudes toward persons with dementia. Scores on the empathy quotient short form (EQ) ranged from 15 to 60. Positive and negative affect ranged from 0 to 50 each, future time perspective ranged from 7 to 70, with 50 being the cut-point for limited versus expansive future time perspective. For psychological flexibility measured by the AAQ-II, scores ranged from 7 to 49. Finally, the mindfulness attention awareness scale (MAAS) scores ranged from 15 to 90.

*p < 0.05, **p < 0.01, ***p < 0.001 for significance of Time X course interactions.

### BATL student learning outcomes: quantitative findings

#### Attitudes

Multivariate analyses of variance revealed a significant time X course interaction [Wilks’ lambda = 0.476, *F*(6, 706) = 52.81, *p* = 0.000] demonstrating improved attitudes toward older adults generally, people with dementia specifically, and community service among BATL students in comparison with Psychology of Aging students or Intro Psychology students (see Table 2). Specifically, BATL students demonstrated improved attitudes toward people with dementia relative to students in Psychology of Aging or PY 101 (Introduction to Psychology). Attitudes toward older adults generally and toward community service also were improved in BATL students relative to Psychology of Aging or PY 101 (see Table 2). Importantly, students in BATL reported greater gains in interest in working with people with dementia.

#### Empathy and mindfulness

Multivariate ANOVA revealed a significant time X course interaction [Wilks’ lambda = 0.842, *F*(4, 348) = 15.67, *p* = 0.000] demonstrating increased empathy but not mindfulness in comparison with Psychology of Aging students or Intro Psychology students. Specifically, BATL students reported greater increases in empathy relative to students in Psychology of Aging or PY 101 (see Table 2).

#### Positive and negative affect and time perspective

Multivariate ANOVA revealed a significant time X course interaction [Wilks’ lambda = 0.479, *F*(8, 562) = 31.22, *p* = 0.000], with greater positive affect reported by students in the experiential BATL class in comparison with students in Psychology of Aging or Introductory Psychology (see Table 2). BATL students also reported significantly less negative affect in comparison with students in Psychology of Aging or Introductory Psychology. Finally, BATL students reported a more open-ended perspective of future time than students in Psychology of Aging or Introductory Psychology. Indeed, Intro Psychology students reported a limited perspective of future time at the end of the semester as their average score fell below the cut-point of 50 (see Table 2).

#### Summary of quantitative findings

In summary, the experiential learning component of the BATL class resulted in improved student learning outcomes relative to a course at a similar 300-level with similar didactic content but no experiential learning component (Psychology of Aging) or a general Introductory Psychology course. These survey findings are strongly supported through in-depth qualitative analysis of BATL student weekly journal entries.

### BATL student learning outcomes: qualitative findings

The qualitative coding team (LBP, BCT, JBT, KDC, and RSA) discovered four themes and six subthemes in the student journals: (1) *attitudes* with subthemes of (a) toward older adults; (b) toward people with dementia; (2) *relationship building creates existential awareness and self-development* with subthemes of (a) relationship building process; (b) existential awareness and self-development; (3) *art fosters social interaction and mindfulness* with subthemes of (a) interaction with art; (b) meaningful social interaction, and (4) *perceived program effectiveness*. Notably, the themes that emerged in this sequential mixed methods design reflect and elucidate the quantitative findings from survey data (Creswell, 2014). See Table 3 for definitions of themes and exemplar quotes.

**Table 3:** Themes, subthemes, definitions, and quotes.

Themes	Subthemes	Definition	Exemplar quotes
Attitudes		A determined way of feeling or thinking about a certain topic or situation, which is typically shown through one’s behavior.	
	Toward older adults, generally	A determined way of thinking or presumed assumptions about older adults that arise pre- and post-BATL experience.	Having to realize that you need help is not as easy. This is why I think many older people are not getting the help they need. They are afraid because no one wants to admit they are unable to perform simple tasks (J2, Spring 2016).
	Toward PWD	A determined way of thinking or presumed assumptions about PWD that arise pre- and post-BATL experience.	They are still living people that enjoy human interaction and want to learn the stories of others, as well as, share their knowledge with you (J4, Fall 2016).
Relationship building creates existential awareness/self-development		Strengthening the relationship between PWD and students will create a greater sense of perspective and self-awareness for students.	
	Relationship building process	The development of effort, trust, and empathy in the relationship between PWD and students.	I was ecstatic though when [PWD] hugged me, and it makes my heart so happy to be getting to know him better and strengthening our friendship (J7, Fall 2017).
	Existential awareness/self-development	The sense of perspective, self-awareness, and gratitude for choices in life.	We were on a high from our experience with these women, because not only are we attempting to provide our participants with life-giving interactions, but we are truly growing and developing in our own personhood as well (J3, Fall 2016).
Art fosters social interaction and mindfulness		The techniques by which art serves as a method for stimulating the mind and emotional expression.	
	Interaction with art	The methods used by students and PWD to share art development as a means of greater understanding and trust for one another.	Mr. Emory turned the photo over, where an advertisement with a bikini-clad woman on the beach caught his eye. “She’s prettier than the lighthouse,” he said. Everyone laughed, and I really enjoyed that moment we all shared (J2, Spring 2018).
	Meaningful social interaction	The life lessons and stories shared by PWD and students that was fostered using art.	I asked if [PWD] was feeling okay, and he said, “Not really, but I think you all will make me feel better (J5, Spring 2016).”
Perceived program effectiveness		The methods in which students deem the class to be an effective method for increasing empathy and understanding.	

#### Attitudes

##### Older adults, generally

Just as in the quantitative attitude survey (see Table 2), BATL students reflected about having pre-conceived and ageist attitudes toward older adults at the beginning of the semester. They stated that they expected to find older adults in a place that was dull and smelled poorly. However, upon first encountering the environment of the adult day service center and increasingly across the semester, it was stated in student journals that the environment of the adult day center differed from previous experiences students had with older adults in care settings. They were pleasantly surprised to be surrounded by bright colors, artwork by clients of the adult day center, and delightful smells that traveled throughout the building.

“I assumed it was going to be another dreary, hospital-like environment where patients lay in bed all day with few stimulating activities, but I was greeted by brightly colored artwork on the walls and a delightful smell that wafted through the whole building (J1, Fall 2016).”

“I was unsure what to expect. I had envisioned something that would resemble the stereotypical nursing home – an antibiotic scent that hits you as soon as you walk through the door, cheap furniture, and a sad, unhomelike atmosphere (J1, Fall 2017).”

The students also expressed surprise toward changed attitudes about the older adults. There was admittance of how students expected older adults to behave; however, seeing them in the atmosphere of the adult day center led to a newfound respect and understanding of their lives as *people*, just like the students themselves. The students also admired how the older adults continued to show gratitude despite the many daily challenges due to living with dementia.

“What a moment for me, to realize that I am surrounded by people who have every right to complain and yet complain less than I do on a daily basis. Their ability to smile despite the circumstances is nothing short of inspiring (J7, Spring 2016).”

“I have to admit that I have been guilty of judging elderly people for their driving or seemingly odd behaviors. I never considered the many things that elderly people struggle with on a daily basis (J2, Fall 2016).”

##### People with dementia

Similarly, a subtheme of changed attitudes toward persons with dementia emerged in the student journals across the semester, akin to the significant time X class interaction in the survey data revealing more positive attitude change toward persons with dementia among students in BATL relative to introductory psychology or psychology and aging students. Across time, BATL students expressed delight to see that their preconceived notions were false and that interactions with people with dementia were enriching. For some students, initially negative attitudes were attributed to a lack of knowledge and experience with the dementia. BATL students expressed surprise and pleasure as they learned the life stories of the people with dementia with whom they were interacting through art. Many students expressed that they learned valuable life lessons from the narratives provided by people with dementia.

“These individuals, while they have bouts of forgetfulness, are still very mentally aware. No matter the mental state, they will always need to be treated with respect because they are still humans with a beautiful unique soul (J1, Spring 2016).”

“I had this preformed image of low functioning patients who were unable to express themselves. To my pleasant surprise, they were very expressive and seemed to formulate their thoughts well (J3, Fall 2016).”

“I think that elderly people in general have so much knowledge they can share with us. Surprisingly, I think people with Alzheimer’s almost have even more to share (J3, Fall 2016).”

The students expressed gratitude for the opportunity to learn and interact with people with dementia. They also emphasized an understanding of how frustrating it can feel for people with dementia to live their daily lives.

“Being in this simulation made me realize I was still highly unaware of just how much struggling Alzheimer’s patients have to do on a daily basis to just complete normal tasks. I cannot imagine having to live every day like this (J2, Fall 2015).”

“I am grateful that I got the opportunity to walk in the shoes of those who have dementia. I think the tour will allow me to have even more empathy with the participants because now I have a better understanding of the hardships they face (J1, Spring 2018).”

Students also expressed how they saw working with people with dementia in producing art through BATL as an opportunity to not just grow mentally but emotionally. They began to see, understand, and digest the life stories and narratives that people with dementia had to offer.

“My mind already knew that people with Alzheimer’s or other dementias are suffering, but now my heart understands too (J1, Fall 2017).”

“They are still living people that enjoy human interaction and want to learn the stories of others, as well as share their knowledge with you (J4, Fall 2016).”

“If I have learned anything, it is that [people with dementia] is just as beautiful of a human being now as she was before she had dementia (J9, Fall 2015).”

### Relationship building creates existential awareness and self-development

#### Relationship building process

Students expressed in the beginning of each semester that people with dementia were hesitant and sometimes reluctant to share personal information with them. They showed an understanding of this reluctancy and a desire to help people with dementia feel that they could confide in the students.

“[people with dementia] appeared to be a little timid when she first walked into the room, which is understandable considering she was surrounded by people she had never met and there was a lot of new stimuli (J2, Fall 2017).”

“It’s like she has so much to offer, but she is reluctant to be completely herself, whether it’s out of fear or distrust or maybe she’s just afraid of being too vulnerable. And in that way I relate to her a great deal (J3, Spring 2015).”

Following this experience of hesitancy, students showed an increased understanding of how to relate to people with dementia and helped them feel as though they could trust each other. They put themselves in the position of people with dementia and met them by means of their life story to help them feel valued and understood.

“I proceeded to ask her in Spanish and told her it was completely her decision. Her eyebrows raised and she began selecting markers and colors to use. It was the realization that someone was committed that they would be willing to speak Spanish (J4, Fall 2016).”

“I am grateful, however, because [people with dementia] reduced motor skills in her hands allowed us to form a community through touch. This truly helped us to bond on a more intimate level than we had previously (J6, Fall 2015).”

Then, as interactions continued across each semester, people with dementia began to become less reserved and share more of their life with the students. The relationship tends to unfold between people with dementia and the students once they begin to see one another as friends rather than simply participants in a class.

“Those topics are his favorite things to discuss when doing art, but each week, he dives a little bit deeper and past the surface level (J4, Spring 2018).”

“In the beginning, they were reserved, understandably, but over the past month they have shared more and more aspects of their personalities with us (J5, Spring 2015).”

“I feel now that I am on a more personal level with [people with dementia], that even though he may not remember exactly who I am each week, he knows I am someone he can trust (J6, Spring 2016).”

Upon approaching the conclusion of each semester, students were able to see the outcome of developing a relationship with people with dementia. They noticed the human connection they made, and they could feel that connection in their hearts through expressions of excitement, accomplishment, and joy. They recognized that it did not matter if their participant’s mind remembered them or not, because their heart did. This qualitative theme also is expressed in the increased positive affect and decreased negative affect found in the quantitative surveys (see Table 2).

“[People with dementia] reiterated many times how she enjoys every minute of our time together. She remembers us. I do not know how well she does, but somewhere inside of her there is a switch, and she remembers (J7, Fall 2016).”

“I do not think that [people with dementia] remembers past weeks with us when he sees us, but I do think that his subconscious remembers which is why he’s always so open about sharing a little more of his life with us each week (J4, Spring 2016).”

“He became so comfortable with himself and me and the art that he began bossing me around, telling me to ‘blot here’ and ‘blot there’ and ‘get this’ as his ink floated all across his artwork (J6, Spring 2016).”

#### Existential awareness and self-development

Students emphasized how when they first began BATL, they saw parts of themselves in which they needed to grow. After learning the core components of the course, they were able to empathize with people with dementia. This helped the students to realize that they really are no different than the participants with whom they were building a relationship. In fact, some students even recognized how they might 1 day grow old and end up in a similar position as their participant, and they hoped someone would be willing to listen to their life story the same way they listened to their participant. This qualitative theme reflects the more expansive perspective of future time and increased empathy found in BATL students in comparison with undergraduate students in introductory psychology or psychology of aging (see Table 2).

“It was then I realized that my trepidation had far less to do with meeting [people with dementia] and more to do with what I might find out about myself. Failure on my part to create a positive experience would mean the real deficit is mine (J3, Spring 2016).”

“And I hope that for the participants’ sake that I can manage to live more mindfully. I believe that practicing more intentional mindfulness will be a good challenge for me (J2, Spring 2015).”

“Everyone grows old eventually, and I can only hope that when that day comes, someone will try to understand my struggles and frustrations as well (J2, Fall 2016).”

Later in the semester, students described the need to put themselves in the position of people with dementia. Students felt as though they could not fully grasp the depth of the struggles these individuals face without being able to see first-hand through art therapy sessions. For example, one student touched on how they began to think how this disease might 1 day affect them, and they could not find that empathy until building a relationship with people with dementia.

“I think the biggest thing I took away from the experience is that you should never assume you know what someone’s going through until you have felt what they feel, you have been where they have been, or experienced what they experienced (J1, Spring 2018).”

“Seeing the world from the perspective of the disabled allowed me to ask the question ‘What would I want done to make the disability less of a hindrance to my life?’ This seems like a simple question, but a role-reversal was required for me (J8, Spring 2015).”

It is because of this relationship building process that existential awareness and self-development occur. The process leads students to have greater respect and admiration for people with dementia with whom they are working, but they also feel accomplished themselves for developing these relationships. They leave the class with a heightened sense of gratitude and appreciation for the world as they express being truly touched by their newfound friendships with people with dementia. More specifically, some students emphasized how they entered the class wanting to make a difference in the lives of people with dementia, but people with dementia made a difference in the students’ lives as well.

“I’m glad I got to know more about his life story in general and what experiences he had to make him so appreciative of life. I have no doubt that it will make me more appreciative of my own (J5, Spring 2018).”

“[People with dementia] paused and said, ‘Thank you for listening to me.’ By giving him an hour of our time, we made him feel heard and important. If we achieved nothing else during art therapy, I would be proud of that moment (J9, Spring 2015).”

“I was there to validate and support [people with dementia] as the person he is, but that day I think it was [people with dementia] who validated me as a person instead (9, Spring 2016).”

### Art fosters social interaction and mindfulness

#### Interaction with art

At the beginning of the course, students expressed how art made it much easier to interact with their participants, especially those participants who might not be as verbal. Art provided them with an outlet to begin growing a relationship with people with dementia. Some people find silence very unsettling, and silence can be especially uncomfortable when first working with people with dementia in a new environment. Art provided students with a concrete and creative means of diminishing the unease of silence as well as creating a space of comfort for less verbal people with dementia to express themselves.

“As [people with dementia] worked we were able to give them attention that was not as awkward as it would be to just sit across an empty table and stare at them. They are made to feel attended to and involved thanks to the coordination of the art environment (J3, Fall 2017).”

“Because of the noise, one might suspect that less connections can be made this way, but for [people with dementia], who does not express verbal language as well anymore, art allowed her to spend a few moments in deep interpersonal connection (J7, Fall 2017).”

Students then began to realize how the art made conversation flow much easier. The art prompted a plethora of ideas with people with dementia that allowed students the opportunity to create a social aspect alongside the art that fostered their blooming friendships, further developing storytelling and narrative between people with dementia and students.

“It amazed me how much just one project could allow [people with dementia] to open up and remember things that perhaps she had not remembered for a long time (J4, Fall 2015).”

“Sometimes it takes some prodding, especially with [people with dementia] since he does not seem too into the artwork, but it’s easy to connect it to the art. Once you get those questions going, [people with dementia] is more than happy to talk about his childhood (J2, Spring 2015).”

“[People with dementia] spoke about his father fishing with a business partner when he was younger. This discussion was definitely prompted by the art therapy because we had all said that his shaving cream art looked like a pond with a fish in it (J3, Spring 2015).”

With the help of the art, students were able to begin understanding the life story of people with dementia beyond surface-level questions. They began to see the actual personhood of people with dementia, and the lives they had lived up to the point of their friendship. Art facilitated a much deeper relationship between the students and people with dementia as students interpreted and analyzed the artwork they created with people with dementia.

“She moved to draw a six-armed female, in this case representing herself in the role of mother to six boys. There was much joking, lots of laughter, and stories shared as we reminisced about all the sacrifices our mothers made for us (J5, Fall 2016).”

“She also began talking about the paint and said that sometimes parts are smooth and sometimes they are rough which could be a metaphor for her life journey (J6, Fall 2016).”

#### Meaningful social interaction

Student journals highlighted how meaningful social interactions with their participants were fostered through art. They described how they began to develop relationships in which they felt truly connected and attached to their newfound friendship as they realized they were no different from their participants. They no longer focused on the dementia diagnosis but instead celebrating their participant’s personhood.

“Whenever I started acting out the first two lines of the poem, ‘Here’s the church; here’s the steeple,’ [people with dementia] both finished it with me. I realized that I had learned some of the same childhood church activities as these women (J7, Spring 2015).”

“One huge and obvious element is his musical ability, and it’s clear that this is a central part of his life. I feel very lucky that he’s been willing to share it with us. This past week he even sang for us while playing guitar (J3, Fall 2017).”

“I watched her pick at the glue on her hands the entire session. I wanted to help her, but I did not want to make her feel incapable. I would laugh and show her the glue on my own fingers while holding the pumpkin. Teamwork (J7, Fall 2016).”

As students continued to have conversations and build relationships with their participants, heart-felt conversations were shared between the students and people with dementia. It began to unfold how people with dementia trusted students enough to share the most difficult and saddening parts of their life story. They were no longer hesitant to express themselves and instead, saw students as a comfortable outlet to share their life story. In turn, we see how students truly care for these individuals and are touched by their stories.

“I am tearing up thinking about when she said, ‘I love music. I used to play… the harp. But I cannot do that anymore. I also play the piano. Well, I used to… it makes me really sad.’ When she said that it tore at my heart (J3, Fall 2016).”

“He looks at me and says, ‘I have Alzheimer’s. I feel bad that my wife has to take care of me because I am sick. I hope I never have to live a day without her.’ After he finished, it took everything in me not to cry (J4, Fall 2016).”

“At the end of the session, [people with dementia] cried as she told us how overwhelmed she was and how beautiful each of us was. This made me tear up because there was nowhere else I would rather be than right there (J3, Spring 2016).”

By the time people with dementia and students have spent several sessions together, the journals reflect friendship that exists between the two. People with dementia and students are excited to see one another; they have developed trust with one another; they have created a bond, and the students have created memories that will last a lifetime. People with dementia are provided a safe space to tell their life story while students gain a new perspective on life through an intergenerational friendship. Once again, this is reflected in the quantitative findings that BATL students increased in positive affect and decreased in negative affect across the course of the semester (see Table 2).

“When we were about to leave, I told [people with dementia] that I would see her next week and she replied, ‘Yay, I cannot wait!’ Not only did that make my day but also assured me that [people with dementia] considers me as a friend (J8, Spring 2015).

“Earlier in the activity, we asked [people with dementia] what his favorite holiday was and he responded with ‘a day like today’ (J7, Fall 2015).”

“Needless to say, [people with dementia] was doing more than just telling his stories. It was much deeper than that. He was reliving those moments that he treasured in his heart, and he trusted my group enough to let us be a part of his world (J4, Spring 2018).”

### Perceived program effectiveness

For this theme, the team kept a count of journal entries related to program efficacy from the beginning to the end of each semester concluding their work with the people with dementia in BATL. For feelings students expressed, it was found that (1) 28 students described gaining a new perspective toward working with older adults and being forever impacted; (2) 13 students described feelings of greater empathy, gratitude, and respect; and (3) six students described feeling humbled and honored by the opportunity. Simply, the students enrolled in BATL perceived the experience to be effective and life changing, both for them and for the people with dementia (see Tables 2, 3).

In summary, the themes discovered in the student journals indicate that existential awareness fosters mindfulness and empathy that subsequently leads a change from ageist attitudes toward celebrating the personhood of the people with dementia (see Table 3). The findings also show that social engagement facilitated by engagement with art was a strong predictor of intergenerational relationship building. These results suggest experiential learning opportunities are a critical component to student education to create both enthusiasm and respect for working with people with dementia, potentially influencing future career paths.

## Discussion

Results of the current sequential mixed methods study demonstrate the benefits of experiential learning opportunities with people with dementia fostered by art therapy in both quantitative surveys and content analysis of weekly BATL student journals (Creswell, 2014). Both quantitative and qualitative findings support substantial student learning outcomes of (1) increased empathy, improved mood, and the themes of relationship building process and meaningful social interaction; (2) more positive and less ageist attitudes toward older adults generally and people with dementia specifically, directly expressed in surveys and journals; and (3) increased empathy and more expansive time perspective among BATL students in survey data and reflected also in the qualitative theme of relationship building and subtheme of existential awareness and self-development. Clearly, qualitative analysis of the weekly BATL journal entries enriched and deepened understanding of the growth in empathy and understanding that students have toward aging and dementia, and the burgeoning awareness that a diagnosis does not limit people with dementia from living a meaningful life.

It is not surprising that, based on quantitative survey results, BATL students showed increased empathy, improved mood, and more positive and less ageist attitude relative to Psychology of Aging and Introduction to Psychology students given the experiential learning required by the BATL course. BATL students are given the opportunity to learn through development of intergenerational relationships that are not offered or achievable in didactic courses focused on adult development and aging, and certainly not achievable in introductory courses to the field of psychology. The structure of BATL decreases the use of age segregation, discrimination, prejudice, and stereotyping associated with ageism (Windle et al., 2020; Levy, 2009; Palmore, 2005). The stereotypic characteristics related to aging can be weakened and changed among college students through intergenerational relationship building. Experiential learning courses such as BATL should become standard, perhaps even required throughout the nation in institutions of higher learning.

The theme of *meaningful social interaction fostered through art* was a key factor in perceived program effectiveness as reported by BATL students. Other themes such as *attitudes toward older adults, generally* and *attitudes toward people with dementia* are centered around the experiential activity of relationship building, fostered by art therapy. Changes in attitudes are conditional for motivating students to see the personhood and digest the life story of people with dementia and pursue careers working with the geriatric population. These experiential opportunities can provide students with a hands-on setting that develops ‘career-ready’ attributes (Spanjaard et al., 2018), and when specifically applied to the geriatric community can highlight how more of these interactions will occur with our aging population (Correia et al., 2019). Indeed, experience with older adults, either personally or professionally, influences career trajectories and facilitates future career paths that involve working with older adults (Dorman et al., 2021; Merz et al., 2017; Moye et al., 2019; Strong et al., 2021). Therefore, it is reasonable to conclude that experiential learning opportunities such as BATL are critical regardless of the career background or intent of students. It is also reasonable to conclude these experiential learning opportunities would not have provided as great an impact without the use of expressive arts-based programs as a social scaffold and bridge, as evidence through student journal entries.

Our study further highlights how intergenerational relationships fostered through art and social interaction rid students of stigma and ageist attitudes toward people with dementia, once again reflected in our mixed methodology. Previous research (Corrigan and Watson, 2002; Kane et al., 2018; Jongsma and Schweda, 2018) has highlighted many of the setbacks emerging adults and people with dementia can possibly face as a result of stigma, such as prejudice and infantilization. The exposure and relationships built through the BATL experiential learning course bring awareness to the personhood and life story narrative that should be shared by people with dementia rather than fore fronting the biological aspect of the disease. Experiential learning decreases the aversion and avoidance emerging adults may experience toward older adults, particularly those living with dementia (Cheng et al., 2011; Low and Purwaningrum, 2020). Direct interaction, facilitated concretely through art therapy and life story, fosters empathy that subsequently diminishes ageist attitudes.

Notably, in the beginning of each semester BATL students recorded in their journals that social interaction with people with dementia could be fostered through concrete art therapy directives. Previous studies have explored several of the benefits surrounding art and music in dementia care (Chancellor et al., 2014; Emblad and Mukaetova-Ladinska, 2021; Koger et al., 1999; Lam et al., 2020), including engaged attention, increased social behavior, and increased quality of life. While these components of BATL are critical, it is important to note BATL is built on the foundation of personhood. It teaches students to embrace the life stories of people with dementia as who they are in the present moment, rather than reflecting on ‘who they used to be.’ Therefore, the emerging themes of *relationship building leading to existential awareness and self-development* and *meaningful social interaction fostered through art* would not be possible without building upon the personhood foundation of the course.

As demonstrated by our study, intergenerational, experiential learning programs that use expressive arts therapies as an interactive tool in healthcare education facilitates self-development and growth among students intending to pursue career paths in healthcare. BATL, a program utilizing art therapy, life story and intergenerational relationships is a course that should be offered nationally and explored with other student learners at various stages and in various fields of study. The results of this study support the impact of experiential learning courses that utilize expressive arts-based programs such as BATL via increased empathy, diminished stigma of aging and dementia, and improved attitudes toward community service.

Additionally, the use of intergenerational, experiential learning has the potential to contribute to social justice concerning aging in the future of healthcare through decreasing ageist attitudes and beliefs. As evidenced throughout, experiential opportunities for intergenerational interaction created more positive views and greater understanding of aging and people with dementia, specifically. Thus, it is within reason to conclude that these interactions also deter ageist viewpoints and discrimination against people with dementia. Consequently, this study highlights the need for further utilization of intergenerational interaction through experiential arts-based programs to grow empathy and lessen the stigma associated with aging and dementia as well as other neurodivergent diseases.

### Limitations

As with any research, the present study has limitations. Of note, the selection bias inherent in the quantitative comparison of students in BATL to those in Psychology of Aging and Introductory Psychology is endemic to this research and findings should be interpreted with this limitation in mind. Second, interpretation of the current results must consider the lack of diversity in region. BATL is currently only offered in two states: Alabama and Illinois. An increase in the demographic reach of BATL may spread its impact on pre-healthcare students’ empathy and attitudes across the nation. To decrease the likelihood of social desirability bias faced by quantitative survey research, future analysis of additional geographically dispersed student journals based in experiential learning courses in other regions would be helpful.

### Conclusions and implications for practice and future research

Despite these limitations, findings from this study have the potential to incentivize experiential learning course offerings throughout the nation that may facilitate choice of future career paths in health professions involving older adults, and particularly people with dementia. Moreover, an increased dementia care workforce would create positive changes in adult day service centers and other long-term care settings in which people with dementia may receive services. Students in such experiential learning courses may become pioneers for shifting dementia care toward an inclusive, person-centered lens, carrying with them for a lifetime the principles of personhood and anti-stigma learned in their work with people with dementia during class. These outcomes were facilitated by the use of expressive arts-based therapies to enhance the quality and speed of relationship building by providing concrete behavioral scaffolds for meaningful social interaction. It is because of the interaction between art, life story, and intergenerational relationship building that the outcomes noted in quantitative surveys and student journals support our conclusion that experiential learning courses incorporating art therapy should be standard training for practice careers in the health and health-related sciences.

In terms of future research, a study that utilizes experiential learning course content like BATL but with a focus on caregivers of people with dementia could be beneficial. This would allow researchers to understand the impact the course has on not only the relationship between student and people with dementia but also caregiver and people with dementia, as they are often handling the heavy load of care. The impact BATL has on empathy, mindfulness, and interest in service beyond that of college students could be explored by conducting research in this area. In addition, further research should be conducted in a manner that focuses on the impact BATL has on the emotional, physical, and verbal skills of people with dementia (Reel et al., 2022). Reel and colleagues found the social interactions between BATL students and people with dementia were more powerful indicators of program success than specific interactions with art *per se*, but art was still an integral tool to enhancing interactions between BATL students and people with dementia. It would be beneficial to explore the impact BATL has on other individuals involved in maintaining the well-being of people with dementia.

The potential for experiential, intergenerational programs that foster relationship building through expressive arts-based therapies have the potential to reach a scope far beyond the population of this study. BATL is a program that can be implemented in various settings including, but not limited to, healthcare settings, church settings, and high school settings. When considering this, there is ample opportunity to utilize a program such as BATL to create an understanding society that not only acknowledges but embraces the personhood of people with dementia. These intergenerational programs can steadily decrease ageism and discrimination against dementia by teaching impactful person-centered care skills. Implementation of such programs have the potential to enhance social justice and support person-centered culture change in dementia care.

## Data Availability

The raw data for this article is not publicly available due to concerns regarding participant anonymity. Requests to access the datasets should be directed to the corresponding author.

## References

[ref1] American Psychological Association (2018). Washington, DC: APA Dictionary of Psychology.

[ref2] Archibald M. M. (2016). Investigator triangulation: a collaborative strategy with potential for mixed methods research. J. Mixed Methods Res. 10, 228–250. doi: 10.1177/1558689815570092, PMID: 35977765

[ref3] Arnett J. J. (2007). Emerging adulthood: what is it, and what is it good for? Child Dev. Perspect. 1, 68–73. doi: 10.1111/j.1750-8606.2007.00016.x, PMID: 39563407

[ref4] Bard J. T. Chung H. K. Shaia J. K. Wellman L. L. Elzie C. A. (2023). Increased medical student understanding of dementia through virtual embodiment. Gerontol. Geriatr. Educ., 44, 211–222. doi: 10.1080/02701960.2022.2067850 35451921

[ref5] Beard R. L. Fox P. J. (2008). Resisting social disenfranchisement: negotiating collective identities and everyday life with memory loss. Soc. Sci. Med. 66, 1509–1520. doi: 10.1016/j.socscimed.2007.12.024, PMID: 18222581

[ref6] Bienvenu B. Hanna G. (2017). Arts participation: counterbalancing forces to the social stigma of a dementia diagnosis. AMA J. Ethics 19, 704–712. doi: 10.1001/journalofethics.2017.19.7.msoc2-1707 28813243

[ref7] Brown K. W. Ryan R. M. (2003). The benefits of being present: mindfulness and its role in psychological well-being. J. Pers. Soc. Psychol. 84, 822–848. doi: 10.1037/0022-3514.84.4.822, PMID: 12703651

[ref8] Carstensen L. L. Lang F. R. (1996). Future time perspective scale (FTP) [database record]. APA PsycTests.

[ref9] Chancellor B. Duncan A. Chatterjee A. (2014). Art therapy for Alzheimer’s disease and other dementias [review of art therapy for Alzheimer’s disease and other dementias]. J. Alzheimers Dis. 39, 1–11. doi: 10.3233/JAD-131295, PMID: 24121964

[ref10] Cheng S.-T. Lam L. C. W. Chan L. C. K. Law A. C. B. Fung A. W. T. Chan W. C.. (2011). The effects of exposure to scenarios about dementia on stigma and attitudes toward dementia care in a Chinese community. Int. Psychogeriatr. 23, 1433–1441. doi: 10.1017/S1041610211000834, PMID: 21729424

[ref11] Correia R. Klea L. Campbell G. Costa A. (2019). Fostering intergenerational education: an experiential learning program for medical students and older adults. Canad. Med. Educ. J. 11, e74–e77. doi: 10.36834/cmej.69327, PMID: 33062093 PMC7522870

[ref12] Corrigan P. W. Watson A. C. (2002). Understanding the impact of stigma on people with mental illness. World Psychiatry 1, 16–20, PMID: 16946807 PMC1489832

[ref13] Creech A. Hallam S. (2013). A study of the impact of participatory arts on the well-being of individuals with dementia. Arts Health 5, 118–136. doi: 10.1080/17533015.2013.825536

[ref14] Creech A. Hallam S. Varvarigou M. (2013). The effect of group singing on psychological wellbeing: a systematic review of the literature. J. Music. Ther. 50, 307–331. doi: 10.1093/jmt/50.3.307

[ref15] Creswell J. W. (2014). Research Design: Qualitative, Quantitative and Mixed Methods Approaches. 4th Edn. Thousand Oaks, CA: Sage.

[ref16] De Alves L. C. Monteiro D. Q. Bento S. R. Hayashi V. D. Pelegrini L. N. C. Vale F. A. C. (2019). Burnout syndrome in informal caregivers of older adults with dementia: a systematic review. Dement. Neuropsychol. 13, 415–421. doi: 10.1590/1980-57642018dn13-040008, PMID: 31844495 PMC6907708

[ref17] Dinos S. Stevens S. Serfaty M. Weich S. King M. (2004). Stigma: the feelings and experiences of 46 people with mental illness. Br. J. Psychiatry 184, 176–181. doi: 10.1192/bjp.184.2.176, PMID: 14754832

[ref18] Dorman H. R. Strong J. V. Tighe C. A. Mast B. T. Allen R. S. (2021). Geropsychology career pipeline perceptions. J. Clin. Psychol. 77, 90–104. doi: 10.1002/jclp.23035, PMID: 32761867

[ref19] Emblad S. Y. M. Mukaetova-Ladinska E. B. (2021). Creative art therapy as a non-pharmacological intervention for dementia: a systematic review. J. Alzheimer’s Dis. Rep. 5, 353–364. doi: 10.3233/ADR-201002, PMID: 34189407 PMC8203286

[ref20] Gerdner L. A. (2000). The efficacy of a personalized music intervention on the decrease of disruptive behaviors in persons with dementia. J. Music. Ther. 37, 13–28. doi: 10.1093/jmt/37.1.13

[ref21] Green J. Thorogood N. (2004). Qualitative methods for Health Research. Sociol. Res. 9, 177–180.

[ref22] Harris P. B. Caporella C. A. (2018). Making a university community more dementia friendly through participation in an intergenerational choir. Dementia 18, 2556–2575. doi: 10.1177/1471301217752209 29338329

[ref23] Holt R. Thorpe R. (2007). The SAGE dictionary of qualitative management research: participative inquiry and practice. SAGE Diction. Qualitat. Manag. Res., 1–312.

[ref24] Iezzoni L. I. Rao S. R. Ressalam J. Bolcic-Jankovic D. Agaronnik N. D. Donelan K.. (2021). Physicians’ perceptions of people with disability and their health care. Health Aff. 40, 297–306. doi: 10.1377/hlthaff.2020.01452, PMID: 33523739 PMC8722582

[ref25] Jones C. Sutherland M. (2005). Art therapy in dementia care: a review of the literature. Dementia 4, 201–219. doi: 10.1177/1471301205051435

[ref26] Jongsma K. Schweda M. (2018). Return to childhood? Against the infantilization of people with dementia. Bioethics 32, 414–420. doi: 10.1111/bioe.12458, PMID: 30106171

[ref27] Kane A. Murphy C. Kelly M. (2018). Assessing implicit and explicit dementia stigma in young adults and care-workers. Dementia 19:147130121880472. doi: 10.1177/1471301218804727 30322274

[ref28] Kitwood T. (1997). Dementia Reconsidered: The Person Comes First. Buckingham: Open University Press.

[ref29] Kitwood T. Bredin K. (1992). Towards a theory of dementia care: personhood and well-being. Ageing Soc. 12, 269–287. doi: 10.1017/S0144686X0000502X 11654434

[ref30] Koger S. M. Chapin K. Brotons M. (1999). Is music therapy an effective intervention for dementia? A meta-analytic review of literature. J. Music. Ther. 36, 2–15. doi: 10.1093/jmt/36.1.2, PMID: 10519841

[ref31] Kooij D. T. Kanfer R. Betts M. Rudolph C. W. (2018). Future time perspective: a systematic review and meta-analysis. J. Appl. Psychol. 103:867. doi: 10.1037/apl0000306, PMID: 29683685

[ref32] Lam H. L. Li W. T. V. Laher I. Wong R. Y. (2020). Effects of music therapy on patients with dementia-a systematic review. Geriatrics 5:62. doi: 10.3390/geriatrics5040062, PMID: 32992767 PMC7709645

[ref33] Lawrence E. J. Shaw P. Baker D. Baron-Cohen S. David A. S. (2004). Measuring empathy: reliability and validity of the empathy quotient. Psychol. Med. 34, 911–920. doi: 10.1017/S0033291703001624, PMID: 15500311

[ref34] Leary M. P. Sherlock L. A. (2020). Service-learning or internship: a mixed-methods evaluation of experiential learning pedagogies. Educ. Res. Int. 2020, 1–9. doi: 10.1155/2020/1683270

[ref35] Levy B. (2009). Stereotype embodiment. Curr. Dir. Psychol. Sci. 18, 332–336. doi: 10.1111/j.1467-8721.2009.01662.x, PMID: 20802838 PMC2927354

[ref36] Low L.-F. Purwaningrum F. (2020). Negative stereotypes, fear and social distance: a systematic review of depictions of dementia in popular culture in the context of stigma. BMC Geriatr. 20. doi: 10.1186/s12877-020-01754-x, PMID: 33203379 PMC7670593

[ref37] Mace N. L. Rabins P. V. (1981). The 36-Hour Day: A Family Guide to Caring for People Who Have Alzheimer Disease, Other Dementias, and Memory Loss. Baltimore: Johns Hopkins University Press.

[ref38] MacKillop J. Anderson E. J. (2007). Further psychometric validation of the mindful attention awareness scale (MAAS). J. Psychopathol. Behav. Assess. 29, 289–293. doi: 10.1007/s10862-007-9045-1, PMID: 39148022

[ref39] Maximiano-Barreto M. A. Ottaviani A. C. Luchesi B. M. Chagas M. H. N. (2022). Empathy training for caregivers of older people: a systematic review. Clin. Gerontol. 47, 704–715. doi: 10.1080/07317115.2022.2127390 36148523

[ref40] Merz C. C. Koh D. Sakai E. Y. Molinari V. Karel M. J. Moye J.. (2017). The big shortage: Geropsychologists discuss facilitators and barriers to working in the field of aging. Transl. Issues Psychol. Sci. 3, 388–399. doi: 10.1037/tps0000137, PMID: 29308422 PMC5751961

[ref41] Mitchell G. Agnelli J. (2015). Person-centred care for people with dementia: Kitwood reconsidered. Nurs. Stand. 30, 46–50. doi: 10.7748/ns.30.7.46.s47, PMID: 26463810

[ref42] Moye J. Karel M. J. Stamm K. E. Qualls S. H. Segal D. L. Tazeau Y. N.. (2019). Workforce analysis of psychological practice with older adults: growing crisis requires urgent action. Train. Educ. Profession. Psychol. 13, 46–55. doi: 10.1037/tep0000206, PMID: 31131069 PMC6530929

[ref43] Muncer S. J. Ling J. (2006). Psychometric analysis of the empathy quotient (EQ) scale. Personal. Individ. Differ. 40, 1111–1119. doi: 10.1016/j.paid.2005.09.020, PMID: 39465450

[ref44] Noice H. Noice T. (2006). The importance of the arts in dementia care. J. Appl. Gerontol. 25, 314–321. doi: 10.1177/0733464806290422

[ref45] Palinkas L. Horwitz S. M. Green C. A. Wisdom J. P. Duan N. Hoagwood K. (2015). Purposeful sampling for qualitative data collection and analysis in mixed method implementation research. Admin. Pol. Ment. Health 42, 533–544. doi: 10.1007/s10488-013-0528-y, PMID: 24193818 PMC4012002

[ref46] Palmore E. (2005). Three decades of research on ageism. Generations 29, 87–90.

[ref47] Peisachovich E. Kapoor M. Da Silva C. Rahmanov Z. (2023). Twenty-first-century skills: teaching empathy to health professions students. Cureus 15. doi: 10.7759/cureus.36076, PMID: 37065306 PMC10096794

[ref48] Peng X. Wu L. Xie X. Dai M. Wang D. (2020). Impact of virtual dementia tour on empathy level of nursing students: a quasi-experimental study. Int. J. Nurs. Sci. 7, 258–261. doi: 10.1016/j.ijnss.2020.06.010, PMID: 32817846 PMC7424149

[ref49] Pillemer K. Schultz L. (2002). “Evaluation of the student assisted independent living (SAIL) service-learning project” in Elder Care and Service Learning: A Handbook. eds. Seperson S. B. Hageman C. (Westport, CT: Auburn House), 252–260.

[ref50] Potts D. C. (2022). Bringing Art to Life: Reflections on Dementia and the Transforming Power of Art and Relationships. Eugene, OR: Resource Publications.

[ref51] Reel C. D. Allen R. S. Lanai B. Yuk M. C. Potts D. C. (2022). Bringing art to life: social and activity engagement through art in persons living with dementia. Clin. Gerontol. 45, 327–337. doi: 10.1080/07317115.2021.1936737, PMID: 34100338

[ref52] Reifman A. Arnett J. J. Colwell M. J. (2007). Emerging adulthood: theory, assessment and application. J. Youth Dev. 2, 37–48. doi: 10.5195/jyd.2007.359, PMID: 33991389

[ref53] Russell C. K. Gregory D. M. (2003). Evaluation of qualitative research studies. Evid Based Nurs 6, 36–40. doi: 10.1136/ebn.6.2.36, PMID: 12710415

[ref54] Sandelowski M. (2000). Whatever happened to qualitative description? Res. Nurs. Health 23, 334–340. doi: 10.1002/1098-240X(200008)23:4<334::AID-NUR9>3.0.CO;2-G, PMID: 10940958

[ref55] Sandelowski M. (2001). Real qualitative researchers do not count: the use of numbers in qualitative research. Res. Nurs. Health 24, 230–240. doi: 10.1002/nur.1025, PMID: 11526621

[ref56] Spanjaard D. Hall T. Stegemann N. (2018). Experiential learning: helping students to become “career-ready,” [review of experiential learning: helping students to become “career-ready”]. Australas. Mark. J. AMJ 26:163–171. doi: 10.1016/j.ausmj.2018.04.003

[ref57] Strong J. V. Allen R. S. Tighe C. Jacobs M. L. Dorman H. Mast B. (2021). What geropsychology trainees think geropsychologists do and what we actually do: a mixed-methods study. Gerontol. Geriatr. Educ. 42, 277–296. doi: 10.1080/02701960.2019.1697253, PMID: 33939939

[ref58] Sweeting H. Gilhooly M. (2008). Dementia and the phenomenon of social death. Sociol. Health Illn. 19, 93–117. doi: 10.1111/j.1467-9566.1997.tb00017.x

[ref59] Valencia A. M. M. (2022). Principles, scope, and limitations of the methodological triangulation. Investig. Educ. Enferm. 40:e03. doi: 10.17533/udea.iee.v40n2e03, PMID: 36264691 PMC9714985

[ref60] Wackerbarth S. (1999). What decisions are made by family caregivers? Am. J. Alzheimers Dis. 14, 111–119. doi: 10.1177/153331759901400210, PMID: 39578672

[ref61] Wakabayashi A. Baron-Cohen S. Wheelwright S. Goldenfeld N. Delaney J. Fine D.. (2006). Development of short forms of the empathy quotient (EQ-short) and the systemizing quotient (SQ-short). Personal. Individ. Differ. 41, 929–940. doi: 10.1016/j.paid.2006.03.017

[ref62] Watson D. Clark L. A. Tellegen A. (1988). Development and validation of brief measures of positive and negative affect: the PANAS scales. J. Pers. Soc. Psychol. 54, 1063–1070. doi: 10.1037/0022-3514.54.6.1063, PMID: 3397865

[ref63] Wehrmann H. Michalowsky B. Lepper S. Mohr W. Raedke A. Hoffmann W. (2021). Priorities and preferences of people living with dementia or cognitive impairment—a systematic review. Patient Prefer. Adherence 15, 2793–2807. doi: 10.2147/PPA.S333923, PMID: 34934309 PMC8684431

[ref64] Werner P. Davidson M. (2004). Emotional reactions of lay persons to someone with Alzheimer’s disease. Int. J. Geriatr. Psychiatry 19, 391–397. doi: 10.1002/gps.1107, PMID: 15065234

[ref65] Windle G. Caulfield M. Woods B. Joling K. (2020). How can the arts influence the attitudes of dementia caregivers? A mixed-methods longitudinal investigation. Gerontologist 60, 1103–1114. doi: 10.1093/geront/gnaa005, PMID: 32447369 PMC7427486

[ref66] Yamashita T. Kinney J. M. Lokon E. J. (2011). The impact of a gerontology course and a service-learning program on college students’ attitudes toward people with dementia. J. Appl. Gerontol. 32, 139–163. doi: 10.1177/0733464811405198 25474214

